# The Validity of Ultrasound Technology in Providing an Indirect Estimate of Muscle Glycogen Concentrations Is Equivocal

**DOI:** 10.3390/nu13072371

**Published:** 2021-07-11

**Authors:** Julia L. Bone, Megan L. Ross, Kristyen A. Tomcik, Nikki A. Jeacocke, Alannah K. A. McKay, Louise M. Burke

**Affiliations:** 1 Exercise and Nutrition Research Group, Mary McKillop Institute for Health Research, Australian Catholic University, Melbourne, VIC 3000, Australia; meg.ross@acu.edu.au (M.L.R.); alannah.mckay@acu.edu.au (A.K.A.M.); louise.burke@acu.edu.au (L.M.B.); 2 Performance Science, Sport Northern Ireland Sports Institute, Belfast BT37 0QB, UK; 3 Department of Nutrition, Case Western Reserve University, Cleveland, OH 44106, USA; kristyen.tomcik@case.edu; 4 Australian Institute of Sport, Canberra, ACT 2617, Australia; nikki.jeacocke@ausport.gov.au

**Keywords:** carbohydrate loading, creatine loading, vastus lateralis, glycogen depletion

## Abstract

Researchers and practitioners in sports nutrition would greatly benefit from a rapid, portable, and non-invasive technique to measure muscle glycogen, both in the laboratory and field. This explains the interest in MuscleSound^®^, the first commercial system to use high-frequency ultrasound technology and image analysis from patented cloud-based software to estimate muscle glycogen content from the echogenicity of the ultrasound image. This technique is based largely on muscle water content, which is presumed to act as a proxy for glycogen. Despite the promise of early validation studies, newer studies from independent groups reported discrepant results, with MuscleSound^®^ scores failing to correlate with the glycogen content of biopsy-derived mixed muscle samples or to show the expected changes in muscle glycogen associated with various diet and exercise strategies. The explanation of issues related to the site of assessment do not account for these discrepancies, and there are substantial problems with the premise that the ratio of glycogen to water in the muscle is constant. Although further studies investigating this technique are warranted, current evidence that MuscleSound^®^ technology can provide valid and actionable information around muscle glycogen stores is at best equivocal.

## 1. Introduction

The determination of muscle glycogen content is of key interest in sports nutrition due to its roles as a fuel source in athletic performance and a regulator of muscle metabolism and adaptation [1,2,3]. A technique that could achieve reliable and valid measurements, while being inexpensive, portable, and non-invasive, would have an enormous potential for increasing knowledge and enhancing practice, particularly in work involving elite athletes and field conditions. Therefore, there is understandable excitement around the commercialisation of ultrasound technology aimed at measuring muscle glycogen in both laboratory and field situations [4,5]. MuscleSound^®^ (Glendale, CO) uses high-frequency ultrasound technology and image analysis from patented cloud-based software to estimate muscle glycogen content from the echogenicity of the image; this feature is based largely on muscle water content, which is presumed to act as a proxy for glycogen [4,5,6]. It was used in research activities to describe changes in muscle glycogen in response to diet and exercise, as well as being a commercially available tool to guide the preparation and recovery of athletes. Extended applications of MuscleSound^®^ were more recently proposed, including the monitoring of the glycogen status of critically ill patients in hospital intensive care units as a measure of metabolic health [7]. Furthermore, ultrasound techniques are being developed as reliable and valid protocols to monitor subcutaneous fat in athletic populations [8]. Nevertheless, this review will focus on the use of MuscleSound^®^ to assess muscle glycogen within sporting populations and scenarios.

Although validation studies have been published [4,5], incongruous experiences with the use of this tool [9] led us to express concerns about a report on glycogen utilisation during a high-level football match using MuscleSound^®^ technology [10]. We questioned the interpretation of these findings, including acknowledgement of data from our own groups, which refute the validity of the MuscleSound^®^ technique [11]. Rebuttal from the study authors [12], which include co-founders of the technique and commercial company, dismissed our concerns based on assertions that the dissenting studies failed to understand the basis of the use of their tool. Furthermore, they noted that only one contradictory dataset is available in the peer-reviewed literature, while the other study remained an unpublished conference presentation [12].

Given the many uses of muscle glycogen measurements in sports nutrition research (e.g., investigations of strategies to enhance glycogen storage and enhanced understanding of strategies to enhance post-exercise adaptation) and practice (e.g., guiding individual athletes to optimally fuel for competition or achieve different levels of carbohydrate ((CHO) availability for training goals), it is important to discern whether the MuscleSound^®^ technique provides valuable and actionable information or whether it might contribute to misleading research outputs and unsupported training and dietary practices. The aim of this review is to examine the available literature on the use of this ultrasound technology to measure muscle glycogen concentrations in athletic populations. To allow a complete account, we will include the results of the unpublished project, while providing full transparency over these data and the cause of their absence from the literature. Such data are valuable given the small number of studies of muscle glycogen involving MuscleSound^®^ in general, and the independent nature of our investigation. We note, in particular, that our study provides the only comparison between biopsy-derived glycogen measurements and the newer estimated fuel level score provided by the MuscleSound^®^ technology. The estimated fuel level is described in a company “position stand” as the metric by which athletes can be given actionable feedback about the suitability of their diet and exercise practices [13]. Although the original technique, producing an estimate of muscle glycogen content in arbitrary units, was described in two validation studies [4,5], we are unaware of any published work that confirms the reliability and validity of the updated technique which, as described in company literature, is the information provided for real-life uses.

## 2. Brief Overview of Methods to Assess Muscle Glycogen

Muscle glycogen is an important fuel store for exercise, as well as a key regulator of metabolism within the muscle cell [2]. Indeed, the development of methods to measure its presence and location within the muscle cell provided the first major advances in the science and practice of sports nutrition [14]. The first measurements of muscle glycogen were made possible by the introduction of the percutaneous biopsy technique to sports science in the late 1960s [15,16,17]. Subsequent modification of the technique included the addition of suction to increase the size of the sample collected [18] and movement of the location of the muscle site by 2 cm for subsequent biopsies to avoid the artefact of damage from the first [19]. This protocol is still used today, and is considered the “gold standard” for assessment of muscle glycogen stores, while acknowledging the invasiveness of the procedure and its downstream limitations on the subjects and environments in which it might be safely and logistically performed. A muscle sample collected by biopsy (typically 20–200 mg according to the size and type of needle) can be treated with several enzymic, histochemical, or electron microscopy procedures to determine the average glycogen content of mixed muscle, the specific glycogen content of fibre types, or the sub cellular location of glycogen, respectively [20,21,22]. In particular, validation studies showed that glycogen measurements from a homogenated muscle sample, freeze-dried to remove variables such as the presence of connective tissue and fluctuating cell water content, provides a reliable measure of the glycogen content of the whole muscle under study [23].

Notwithstanding the utility of the biopsy technique in allowing an in-depth analysis of glycogen location within and between different muscle fibres, as well as enabling measurement of a vast host of other muscle metabolites, signalling molecules, and other “omic” interests, there is recognition that other techniques are needed to address the practical and ethical concerns associated with its use. The addition of magnetic resonance spectroscopy (MRS) to magnetic resonance imaging, and the increase in the power of the magnetic fields generated by such equipment, has allowed MRS to become an additional tool to indirectly assess muscle metabolites and fuel stores. This technique uses magnetic fields and radio waves to measure tissue glycogen by monitoring either ^13^C natural abundance levels, or ^13^C atoms incorporated into glycogen following the administration of a ^13^C substrate [20]. MRS techniques were shown to provide a reliable assessment of muscle glycogen in healthy and clinical populations [24] and have been applied to assessments of glycogen utilisation or storage in athletic populations [25,26]. Although MRS offers the advantages of providing rapid, non-invasive, and potentially repeated measurements of glycogen in various tissues, its disadvantages include the expense and the need to access specialised facilities and expert technicians.

## 3. MuscleSound^®^ Measurements of Muscle Glycogen

MuscleSound^®^ is a commercially available tool which uses high-frequency ultrasound and patented software to derive an assessment of muscle glycogen. This technique, utilising a point of care device and cloud-based software, offers features that address the disadvantages of both biopsy and MRS-derived assessments of muscle glycogen; namely, it is a protocol that is non-invasive, relatively cheap, rapid, and portable. The original development of MuscleSound^®^ was undertaken by researcher-practitioners who noted apparent correlations between ultrasound images and muscle glycogen content. The image greyscale produced on an ultrasound scan is based on the intensity of the ultrasound “echogenicity”, or reflection of an ultrasound beam, with the beam being both produced and detected by the transducer forming the ultrasound image [27]. Ultrasound beams are reflected at the boundary between two materials with different acoustic impedances, with strong reflections showing as white on the ultrasound image and weaker echoes being grey [27]. Proprietary information within the MuscleSound^®^ software aligns a darker image with greater glycogen stores using the principal that greater glycogen, and its associated water content in the muscle fibre, should reflect the lower echo intensity between soft tissue and water [5,27]. Conversely, in instances when glycogen is low and there is less fluid, the echo intensity is greater due to the increased visibility of other tissue boundaries, thus resulting in a brighter image [5,27].

The MuscleSound^®^ technique, validated in two studies against biopsy-derived measures of muscle glycogen [4,5], produces a glycogen score in arbitrary units (hereafter identified as a.u.), where values are provided in bands of 5, between 0 and 100, with an unknown typical error. However, the assumed relationship between muscle glycogen and water was noted as a technical issue requiring further investigation in one of these foundation studies [5]. Indeed, according to an undated company position stand on the science and application of MuscleSound^®^ located on the company website [13], further developments of the technique recognised scenarios in which muscle glycogen and water deviated from this relationship, and they provided recommendations for situations in which the use of MuscleSound^®^ is considered optimal and those that are considered to be sub-optimal (see Section 6.1). According to this position stand, the current output from the MuscleSound^®^ proprietary software provides a muscle energy status, representing the mean of an estimated fuel level and a muscle fuel rating. The company material describes muscle fuel as predominantly glycogen, with contributions from carnitine, creatine, and protein. The estimated fuel level is determined by “placing an image in context of the maximum (100) and minimum (0) points of glycogen obtained from a bank of images captured for a specific participant”, with the muscle fuel rating providing a separate rating compared to a large databank of images from many athletes [13]. We are unaware of any published validation studies of these new metrics, although the company literature promotes these values for field use in providing athletes with feedback about changes in muscle glycogen resulting from their diet and exercise strategies [13].

## 4. The Bone Study of MuscleSound^®^

In 2014, we became aware of the newly launched MuscleSound^®^ tool and realised both its potential to enhance our work as sports nutrition practitioners and applied researchers, and the opportunity to test its reliability and validity as an additional arm within a pre-existing project investigating interactions between manipulations of muscle glycogen and creatine content [28]. Although the main aim of the study was to investigate the effect of creatine and glycogen loading on cycling performance, we embedded a research arm to investigate artefacts in the measurement of lean mass by dual energy X-ray absorptiometry due to changes in muscle water content associated with changes in muscle creatine, glycogen, and water content [29]. We invited the MuscleSound^®^ group to use this opportunity to further test their technology in scenarios that are very common in sports, but outside the conditions under which their own validation studies were conducted [4,5]. They were not involved in the study design, funding, or conduct; rather, we funded their visit to Australia to train us in the use of their technique. Although we had intended to use our own ultrasound equipment to capture images, IT security requirements at our workplace prevented us from uploading images to a cloud-based server. Therefore, MuscleSound^®^ loaned us an ultrasound machine and provided complimentary results of muscle glycogen estimates derived from their proprietary software, using the original algorithm, for the duration of the study. A contract was signed to oversee return of the equipment and, on completion of the study, the contribution of our data towards further development of the proprietary algorithm. Following data collection, the equipment was returned, and a poster presentation was prepared for the 2016 annual meeting of the American College of Sports Medicine (ACSM).

In the days prior to the ACSM meeting, we received a directive from a legal firm engaged by MuscleSound^®^ to withdraw the poster from the conference. Although we complied, an electronic version of the abstract was included in a review of methods to assess muscle glycogen in sports nutrition activities without our involvement [20]. To find a path to our ethical obligation to be transparent with all research outcomes via peer-reviewed publication, we subsequently agreed to allow the company to re-analyse the scans from our study using an updated algorithm and interpretation framework. However, recognising the commercial sensitivity of the two datasets, we decided to delay publication until other studies of the validity of the technique, undertaken by independent research groups, but partially funded by MuscleSound^®^ [9], were released. Although the main results of the current study, that MuscleSound^®^ failed to provide valid estimates of muscle glycogen, are no longer original, the additional range of scenarios that we have studied (supervised CHO loading, prolonged exercise depletion, and the addition of creatine loading) present novel outcomes. Furthermore, they include the only direct comparison of biopsy-derived glycogen assessments and the MuscleSound^®^ estimated fuel level score, which is promoted in company material [13] as a commercially available use of this tool to provide actionable information to athletes about the suitability of their diet and exercise activities. Once published, we will provide the original data to the MuscleSound^®^ company.

### 4.1. Overview of Study Methods 

**Figure 1:**
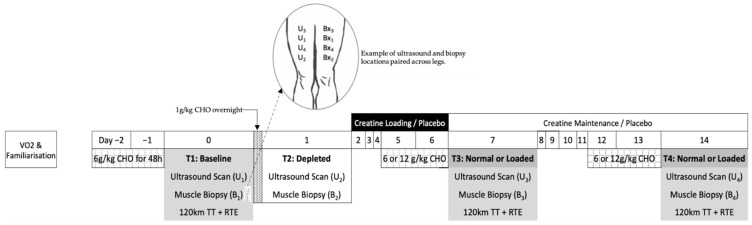
Overview of study design. (TT, time trial; RTE, ride to exhaustion; ultrasound; and Bx, biopsy).

Twelve competitive male cyclists participated in this study, which was approved by the human research ethics committees of the Australian Institute of Sport (20140612) and the Australian Catholic University (2014 254N). These subjects (32.6 ± 5.1 years; 79.2 ± 9.5 kg; 5.1 ± 0.6 L/min maximum oxygen consumption, and 639 ± 115 W maximum power output) represented a sub-group of a larger cohort who undertook the main project under which this study was performed [28]. This study employed a parallel group design to investigate the effect of creatine loading, followed by a within-group cross-over application of carbohydrate loading on muscle substrate, water content, and performance (Figure 1). The participants came in for four separate biopsy and ultrasound measurements; baseline (day 0, 6 g carbohydrate/kg body mass (BM)/day for 48 h), glycogen depleted (day 1) and either glycogen loaded or glycogen normal (6 g carbohydrate /kg BM/d for 48 h) with or without creatine supplementation (days 7 and 14). Manipulations of creatine and glycogen stores were achieved by implementing “best practice protocols” of creatine loading (20 g/day for 5 days loading and 3 g/day for maintenance) [30] and glycogen loading (12 g CHO/kg BM/day for 48 h) [31] through a standardised pre-packaged diet. Furthermore, a supervised cycling protocol of ~3.5 h was undertaken to deplete muscle glycogen stores. The cycling protocol involved a 120 km time trial, with alternating 1 km and 4 km sprints every 10 km, followed by a ride to exhaustion at 8% gradient and 88% VO_2_max; further details can be found in [28]. Participants consumed 60 g/h CHO during the cycling protocol with post-exercise intake of a low CHO diet (<1 g CHO/kg BM) to restrict the repletion of glycogen stores before a further assessment of muscle glycogen content the following morning. This protocol was chosen to allow us to align our assessment of the depleted glycogen condition with DXA-estimates of body composition assessed according to best practice protocols (overnight fasted and rested conditions [32]). This study design provided situations where muscle glycogen was measured under baseline and normalised conditions, a depleted condition, and CHO loaded with or without creatine loading. Four biopsies were conducted over the course of the study, with each being collected from the same leg from an incision that was as least 2 cm from the previously biopsied site [19] [Figure 1: Bx_1_–Bx_4_]. The protocol used for these biopsies, and the determination of glycogen and creatine content in the muscle samples, is described in full elsewhere [28].

### 4.2. MuscleSound^®^ Score (2015)

Two of the researchers involved with this study were trained to capture ultrasound images on the vastus lateralis using a portable ultrasound machine. They practised this technique to achieve acceptable reliability with repeat images and were assigned to the study roster so that each participant was scanned by a single technician over the duration of their study involvement. Thereafter, on each occasion that a muscle biopsy was performed, ultrasound images were captured using the same machine (Terason T3000, TeraTech Corporation, Burlington, MA, USA) and by the same technician. Five ultrasound images were captured at each time-point on the vastus lateralis of the contra-lateral leg, tracking the location of the specific incision on the biopsied leg (Figure 1: U_1_–U_4_). A further five images were captured on the contra-lateral leg at the site of the original (baseline) assessment (Figure 1: U_1_). The protocol followed MuscleSound^®^ guidelines with images captured on the transverse plane at a depth of 4 cm and a gain of 45 with the muscle relaxed. The transducer head was manipulated to achieve a bright fascia, which defined the muscle boundary for the region of interest. Images were then uploaded to the MuscleSound^®^ software (v.2015, MuscleSound^®^, LLC, Denver, CO, USA) and processed according to their proprietary protocols. The MuscleSound^®^ score (0–100) was subsequently provided in arbitrary units (a.u.), noting that such scores were provided in bands of 5 a.u. [5]. A single score used in the statistical analysis was obtained by averaging the score from each of the five images at each site.

### 4.3. Estimated Fuel Level (2017)

The ultrasound images were re-analysed by MuscleSound^®^ using an updated protocol titled “estimated fuel level”. Details of this proprietary process were not published, but are described as “placing an image in context of the maximum (100) and minimum (0) points of glycogen obtained from a bank of images captured for a specific participant [13]”. We have described these values as “points” to distinguish them from the original metrics (described as a.u.).

### 4.4. Statistical Analysis

Agreements between biopsy and MuscleSound^®^ estimated fuel level scores were assessed by 95% intra-class correlation based on a one-way, consistency model [33]. Pearson’s correlations were used to determine the association between site-specific measurements. The muscle glycogen concentration across the different states was assessed using a general linear mixed model (LMM) using the R package lme4 (R Foundation for Statistical Computing, Vienna, Austria). All models included a random intercept for subject to adjust for baseline levels and inter-individual homogeneity. Additionally, creatine dry weight was included as a covariate in all models. Each model was estimated using restricted maximum likelihood, with the tests for statistical significance of the fixed effects performed using type II Wald tests with Kenward–Roger degrees of freedom. Where significant fixed effects were evident, Tukey’s post hoc comparisons were performed to detect specfic condition differences.

### 4.5. Results

**Figure 2:**
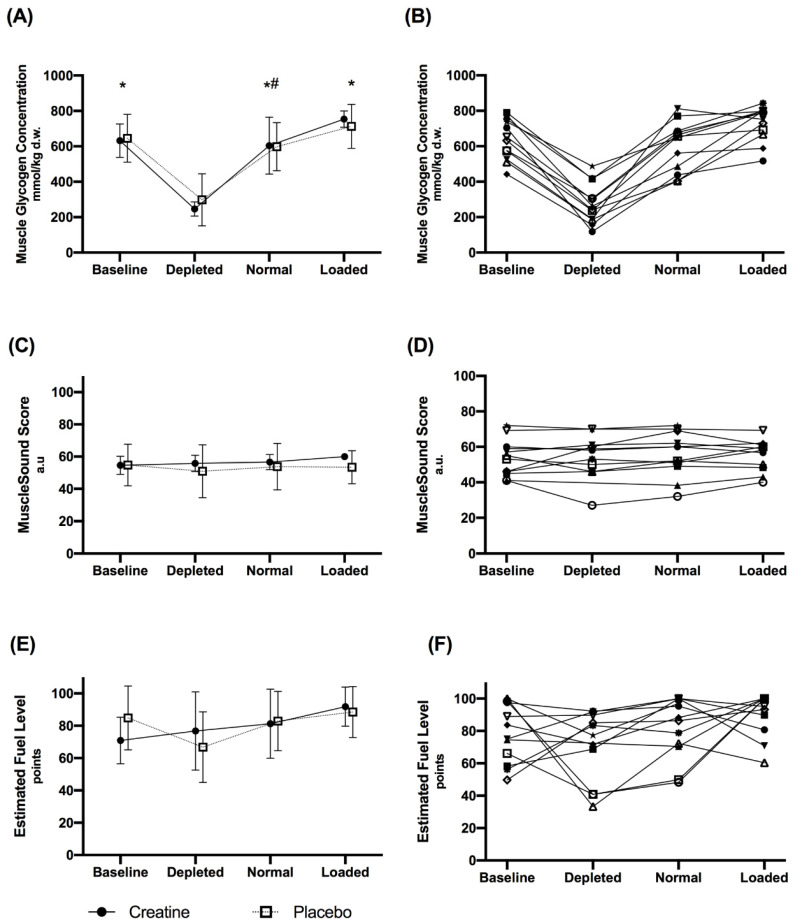
Biopsy-derived muscle glycogen concentrations (**A**,**B**) MuscleSound^®^ scores in arbitrary units (**C**,**D**) and estimated fuel level points (**E**,**F**) for the creatine and placebo conditions during each dietary treatment: baseline, glycogen depleted, normal and carbohydrate loaded. Values are mean ± SD in panels A, C and E with individual data shown in panels B, D and F. * Indicates a significant difference to depleted. # Indicates a significant difference to loaded.

The effects of the different dietary treatments on biopsy-derived muscle glycogen concentrations, MuscleSound^®^ scores and estimated fuel level points, and the influence of placebo and creatine supplementation are summarised in Figure 2. These data represent measurements taken on one leg using the established protocol to site sequential muscle biopsies (B_1_–B_4_), with the MuscleSound^®^ score being taken on the contra-lateral leg at the corresponding site (U_1_–U_4_). There was a significant main effect for the different dietary treatments on biopsy-derived muscle glycogen concentrations (F(3,29) = 61.2, *p* < 0.001). Values of biopsy-derived glycogen concentrations for glycogen depletion were lower than baseline, CHO loaded, and normal conditions (*p* < 0.001), while values for carbohydrate loading were significantly greater than normal (*p* = 0.013). Since there were no differences in muscle glycogen between the creatine and placebo groups (F(1,10) = 0.1; *p* = 0.760), a combined mean value for the results for each treatment was derived. Nevertheless, creatine dry weight was a significant variable within the model, indicating that higher creatine dry weight values were associated with increased muscle glycogen content (F(1,33) = 8.6; *p* = 0.006).

There were no differences in MuscleSound^®^ scores between dietary treatments (F(3,27) = 1.1; *p* = 0.384) or between the placebo and creatine groups (F(1,10) = 0.3; *p* = 0.627). Furthermore, no statistically significant differences between dietary treatments (F(3,28) = 1.1; *p* = 0.352) or between the placebo and creatine groups (F(1,10) = 0.2; *p* = 0.701) were evident for the estimated fuel level points. Finally, creatine dry weight was not associated with either MuscleSound^®^ (F(1,28) = 2.04; *p* = 0.165) or estimated fuel level points (F(1,37) = 3.07; *p* = 0.088). An ICC of −0.75 (95% CI −0.85, −0.59) was apparent in the relationship between biopsy-derived muscle glycogen content and MuscleSound^®^ scores, with a similarly unclear relationship between biopsy−derived muscle glycogen content and estimated fuel level points (ICC of −0.72 (95% CI −0.83, −0.55)). The estimated fuel level points were quantitatively higher than the MuscleSound^®^ score, reflecting an amplification of the original values from an absolute value to a relative range.

**Figure 3:**
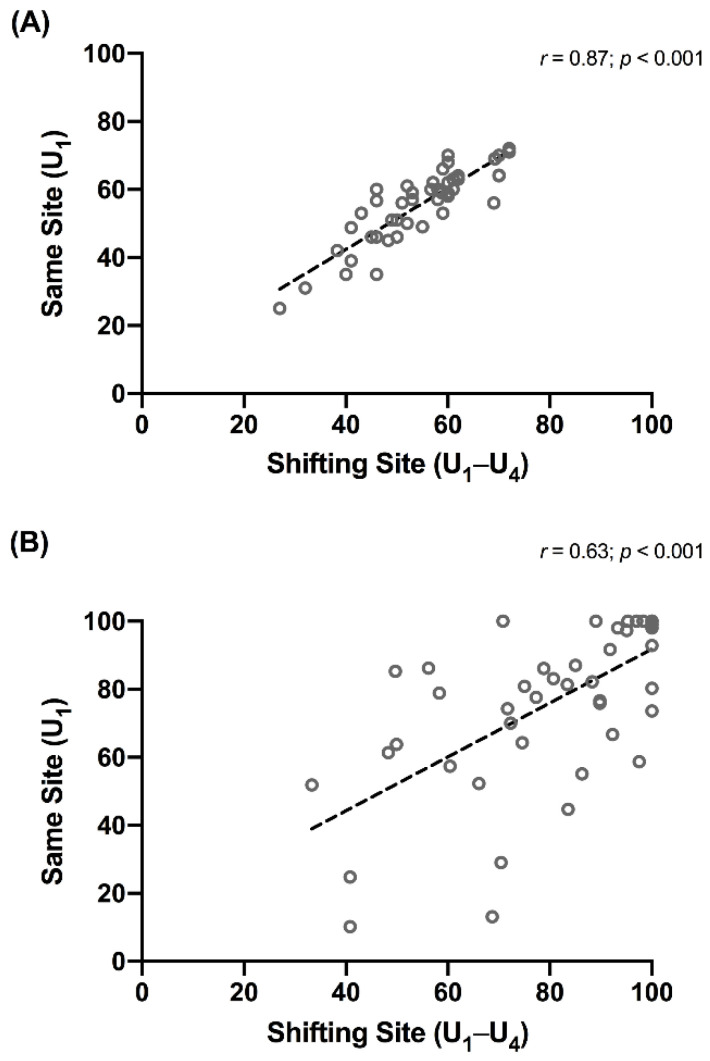
Correlation between values for MuscleSound^®^ score (**A**) and the estimated fuel level (**B**) measured at the same site (U_1_) and at the shifting site (U1_1_–U_4_) corresponding to the muscle biopsy.

There were no differences between the MuscleSound^®^ score values collected from the site on the contra-lateral leg corresponding to the biopsy site (U_1_–U_4_) and the measurements taken on a static site of the leg (U_1_) for each treatment. Indeed, there was a significant correlation (*r* = 0.87 (95% CI: 0.78–0.93); *p* < 0.001) between the values from the two different sites (Figure 3A). Likewise, there were no between-site differences in the values of estimated fuel level points for each treatment, but the correlation between these values was lower (Figure 3B; (*r* = 0.63 (0.42–0.78); *p* < 0.001)). A separate examination of the results of MuscleSound^®^ scores taken at the same site (U1) showed a small but significant (*p* = 0.024) difference between depleted (53 ± 13 a.u.) and loaded treatments (57 ± 10 a.u.). However, these did not differ from baseline (56 ± 3 a.u.) or normal (57 ±12 a.u.) values. Furthermore, the numerical difference between the mean values was smaller than the gradation (bands of 5 a.u.) between sequential results. The estimated fuel level points mirrored these outcomes with an increased spread in both the mean values and SD. Differences were detected between depleted (59 ± 30) and loaded (91 ± 14) treatments (*p* = 0.026), but neither of these differed from the baseline (78 ± 19) or normal (77 ± 22) values.

## 5. The Literature Involving MuscleSound^®^ Assessment of Muscle Glycogen 

A summary of the available literature in which MuscleSound^®^ technology was used to assess changes in muscle glycogen content resulting from dietary and exercise interventions is provided in Table 1. This includes the validation studies, which originally introduced the use of MuscleSound^®^ as a proxy for biopsy-derived measures of muscle glycogen [4,5], two other data sets in which muscle glycogen content and its changes were assessed by ultrasound and chemical protocols [9], the Bone data presented here, and a recently published study in which MuscleSound^®^ alone was used to assess changes in muscle glycogen content over an exercise session [10]. A final paper, involving the use of the newer estimated fuel level metric, was not included in this table due to differences in its focus and methodology, but it is included in the discussions.

Although laboratory-based cycling protocols represent the most frequently investigated mode of exercise, several studies have included real-world competition involving field-based team sports (see Table 1). Dietary manipulations include low, moderate, and high CHO intakes, as well as creatine loading. Muscle and body water content, although not directly measured in any of these studies, is likely to be altered by the acute effects of exercise as well as exercise-associated dehydration. Although vastus lateralis was the muscle investigated in the majority of studies, differences in study protocols around the MuscleSound^®^ assessment included muscle tension (relaxed vs. contracted), whether the same or contra-lateral leg was used between or within glycogen-assessment protocols, whether the scan was meant to represent the same or a related muscle site, and how many scans were used to derive the MuscleSound^®^ outcome.

The first two publications involving MuscleSound^®^ were designed to directly validate its use for indirect assessment of muscle glycogen concentrations, measuring glycogen content before and after a 90 min steady-state [4] or ~158 min time-trial cycling protocol [5] at the same or a similar site in the chosen muscle. In both studies, the ultrasound scan and subsequent biopsy were undertaken at the same site, with the ultrasound being conducted first, followed by the collection of the biopsy, guided by the ultra-sound. In the first study [4], one leg was used for the pre-exercise assessment, while the contra-lateral leg was used in the same manner for the post-exercise assessment to avoid the effect of the muscle biopsy on subsequent glycogen storage at that muscle site [19]. In the second study, the same leg was used for both assessments, but the second biopsy was taken at a site 2 cm from the first; this is sufficient to avoid the effects of such muscle damage on glycogen content, at least by the biopsy technique [19]. With the longer cycling protocol, Nieman et al. reported significant correlations between the two measurement techniques for pre- (0.92, *p* < 0.001), post- (0.90, *p* < 0.001), and exercise-associated changes (0.92, *p* < 0.001) in glycogen concentrations in the vastus lateralis muscle [5]. Here, the chemical method showed a reduction in muscle glycogen content by 77 ± 17%, representing an absolute change of ~71 mmol/kg ww (~306 mmol/kg dw) glycogen; the absolute scores on the MuscleSound^®^ 0–100 a.u. rating were not provided [5]. These data represent a more practical and representative examination of glycogen utilisation during a prolonged endurance sport than the earlier study of Hill and San Millan [4], which employed a 90 min cycling protocol and biopsy collection from the infrequently studied rectus femoris muscle. Indeed, in the earlier study, absolute glycogen values achieved by the dietary preparation protocol and their subsequent utilisation during exercise were lower, with muscle glycogen being reduced by 36% according to chemical analysis and a MuscleSound^®^ change score of ~60 to ~40 a.u. (33% decrease). Nevertheless, correlations between the chemical and ultrasound-mediated assessments of muscle glycogen concentration had pre- (0.92, *p* < 0.001), post- (0.90, *p* < 0.001), and exercise-associated changes (0.92, *p* < 0.001) [4].

In contrast to these earlier reports, an investigation of two separate exercise scenarios by another research group failed to find consistency between the MuscleSound^®^ scores and biopsy-derived assessments of muscle glycogen changes due to exercise and diet [9]. In these studies, which involved cycling and a rugby league match, measurements were made on the same leg, with the biopsy sites 2 cm apart [9]. Although the muscle biopsy protocol identified a ~40% reduction in glycogen content as a result of match play in a real-world rugby league competition (pre-game: 443 ± 65 and post-game: 271 ± 94 mmol/kg dry weight (dw), *p* < 0.001), there were no changes in the MuscleSound ^®^ scores (47 ± 6 vs. 49 ± 7, *p* = 0.4).

A separate study, involving a cycling protocol, was undertaken to remove any potential confounding effects associated with the characteristics of rugby play (i.e., intermittent nature and the magnitude of the muscle contractile forces) that might interfere with the ultrasound image and explain the discrepant results. This second investigation involved an exercise-depletion protocol after which either a low carbohydrate diet or a carbohydrate loading regimen was implemented for 36 h [9]. Although biopsy-derived muscle glycogen concentrations after the carbohydrate loading diet were more than doubled in comparison to 36 h of low carbohydrate recovery (~531 vs. 252 mmol/kg dw, Table 1), there were no differences (*p* = 0.9) in corresponding MuscleSound^®^ scores (56 ± 7 vs. 54 ± 6 a.u.). In summary, two separate studies of different types of exercise failed to find significant correlations between changes in muscle glycogen concentration and changes in MuscleSound^®^ scores, and, in both protocols, the ultrasound results failed to detect what could be considered predicable changes in glycogen stores.

The results of the Bone study, presented in this paper, are in agreement with the latter two datasets in finding that the MuscleSound^®^ technique failed to provide meaningful information about muscle glycogen concentrations in athletes. The mean values for muscle glycogen derived from chemical analysis of mixed muscle samples showed larger ranges than reported in the comparative literature, with pre-exercise values after a glycogen loading technique of ~730 mmol/kg wet weight (ww) and a post-exercise reduction of ~364 mmol/kg ww. These values reflect the more aggressive CHO loading regimen and the demanding nature of the exercise protocol. Despite a greater opportunity to detect differences in muscle glycogen, we found that the original MuscleSound^®^ technique generally failed to track the results achieved by chemical analysis of mixed muscle biopsy samples across a range of diet and exercise manipulations, and failed to show the expected significant changes in glycogen concentrations. Individual data showed a range of responses, both in magnitude and direction, in response to each treatment (Figure 2). The only MuscleSound^®^ comparison that yielded a statistically significant difference involved measurements taken from the same site between the depleted and loaded treatments. However, in the case of the original scoring system, the difference was numerically small (53 ± 13 vs. 57 ± 10 a.u.) and was less than the band (5 a.u.) by which results were provided, rendering it of minimal clinical value. Furthermore, this analysis failed to detect differences between the normal glycogen stores and treatments that either increased or decreased these. The estimated fuel level, an updated MuscleSound^®^ metric representing results relative to the lowest and highest scores for the individual athlete, mirrored these results. Although this metric amplified the numerical value of the original score results, and created a greater difference between the mean values, it also increased the range of the results. Therefore, it failed to change the ability of the protocol to detect differences between most treatments.

Two additional publications, which involved the use of MuscleSound^®^ to investigate changes in muscle glycogen in scenarios of real-life sports without alternative confirmation of glycogen stores, are available. One study [10] involved an investigation of changes in muscle glycogen during a football (soccer) match in a professional American league (Table 1). Players followed their typical nutrition practices before and during the match, while the MuscleSound^®^ technique was used to assess glycogen stores pre- and post-game. From the methodology described in the paper, we assumed this protocol involved the traditional MuscleSound^®^ score technique, albeit with results presented as “points”, rather than the new metrics described in the company’s position stand [13]. There was no confirmation of these results with an independent chemical measurement of glycogen, nor was the hydration status of the players measured before or after the match. Nevertheless, the study reported a mean decline in MuscleSound^®^ glycogen scores of 20% over the course of the match, with inter-individual ranges of 6% to 44%, and some variability in the size of the pre-game stores. As predicted, but not verified by information on individual workload characteristics of the specific game, the decline in muscle glycogen points was numerically greater in midfield and forward players than defence players, and was lowest in the goal keeper. Although these results appear unremarkable, the authors suggested that the protocol identified players who had not adequately fuelled prior to the game, as well as players who might undertake more aggressive fuelling strategies during the game. Here, we note that if within-game fuelling provides an additional exogenous fuel source as glycogen stores become depleted, rather than substantially changing patterns of glycogen depletion during the match, the pre- and post-measurement of glycogen by any technique may provide confusing results.

The final publication involved the use of MuscleSound^®^ to monitor resting levels of glycogen in U.S. Division 1 collegiate female volleyball players on each morning of a 9 day pre-season training camp [35]. The MuscleSound^®^ information was provided in the form of muscle fuel rating, which, as previously noted, remains unvalidated in a peer-reviewed published format. This investigation focused on bilateral asymmetries in the glycogen stores in the rectus femoris in these athletes prior to each morning’s training session. The study reported an increase in muscle fuel ratings from the first to second day, with a sustained elevation over the rest of the camp and a 58% difference (higher level) between ratings for the dominant versus non-dominant leg. Although the temporal changes did not track with the training load over the camp (higher in the first days), the authors noted that no dietary control or assessment was implemented. The difference in fuel ratings between legs was attributed to faster rates of glycogen storage in “the more conditioned” dominant leg. Although endurance-trained muscle is known to have higher resting glycogen stores than non-trained muscle (e.g., 500 vs. 350 mmol/kg dw [36]), it is difficult to imagine that the magnitude of difference between legs within the same well-trained athlete would be as large as reported, albeit with a different assessment metric (muscle fuel rating of 52 vs. 33 points). The authors suggested that bilateral asymmetries in glycogen content in volleyball athletes might be used to assess for injury risk, noting that large asymmetries and bilateral deficits in muscle strength are sometimes linked to injuries in athletes [35]. Although this would be a potentially valuable application, there is presently no validation of either the muscle fuel rating score as a measure of muscle glycogen, whether glycogen utilisation patterns are sufficiently different between limbs across a range of symmetrical and asymmetrical exercise activities detected by any technique, nor whether this is associated with injury risk or patterns.

In summary, evidence supporting the use of ultrasound technology, and particularly the MuscleSound^®^ proprietary technique, as a valid measure of muscle glycogen stores is equivocal. In terms of its use as a research tool, two data sets involving laboratory-based cycling protocols validated a correlation with measurements of the glycogen content of a biopsy-derived mixed muscle sample, providing a measure of muscle glycogen from 0–100 in arbitrary units under controlled conditions. Furthermore, the changes in muscle glycogen stores were in line with the expected outcomes of various diet and exercise protocols. Another data set collected in a field setting provided glycogen score results that were logical, but not independently verified. Three other data sets involving lab and field-based uses, however, conflict with these findings. Two collected in cycling models in controlled laboratory conditions, and another undertaken in a real-life team sport competition, failed to find correlations between the two sources of information on glycogen stores. Most importantly, none of these data sets were able to consistently detect differences in MuscleSound^®^ scores despite supervised manipulations of diet and exercise that are known to achieved substantial changes. In one of these studies, a new technique to present MuscleSound^®^ results, described in a company-issued position stand, and presumed to represent its current commercial application, also failed to detect outcomes that would be predicted by the study interventions. This occurred even when undertaken with standardised protocols (e.g., use a single trained tester, laboratory conditions, and the averaging of five separate scans) that might not be possible under the real-life conditions for which it is promoted. Two major issues around the validity and reliability of the MuscleSound^®^ technique have been identified for discussion.

## 6. Validity of the MuscleSound^®^ Technique: The Glycogen: Water Ratio

### 6.1. General Principles

The MuscleSound^®^ technique is based on the principle that the echogenicity or brightness of an ultrasound image reflects the speed of the sound waves reflected by scanned tissues, and in turn, their water content [10,12]. Water, which provides little resistance, produces a dark (hypoechoic) image that can be quantified via the pixel intensity of the image on the scan image [10,12]. In turn, muscle glycogen is quantified by the assumption of a constant relationship with bound water of 1:3 [10,12]. Such calculations are achieved when an image captured by a high-frequency ultrasound is examined by the cloud-based proprietary software of the MuscleSound^®^ company.

Although it is well accepted that fluid is stored when glycogen is formed, the persistence of a fixed relationship over a range of glycogen concentrations has been challenged both in the general literature and in relation to the MuscleSound^®^ protocol [9,10,11,12,37,38]. The first validation study of MuscleSound^®^ [4] did not identify the water to muscle glycogen ratio as an underpinning principle of the ultrasound technique; this explanation was provided in the subsequent validation study. Here, although a tight correlation between ultrasound and biopsy-derived measures of muscle glycogen was reported, the authors noted that “additional research is needed to determine how exercise-induced changes in muscle water content influence this relationship”. Indeed, knowledge of factors that change the muscle glycogen to water ratio, or muscle water content, formed the basis of our recent letter expressing concerns around the MuscleSound^®^ technology [11], wherein we noted that these can change in variable directions as a result of diet-exercise manipulations. The literature on this issue will now be summarised.

Studies on the relationship between tissue water and glycogen content were undertaken in both the liver and muscle in humans and rodents. In the latter case, direct chemical analysis of whole tissues was used to calculate a glycogen:water ratio of 1:2.7 in rat livers under conditions where non-glycogen solids remained constant [6]. However, Sherman et al. [37] failed to find a consistent ratio of glycogen and water in rat skeletal muscle when manipulations to both increase and decrease glycogen content were undertaken. Meanwhile, studies on human subjects are limited to protocols using indirect or sampling measurements. An early investigation of carbohydrate loading [38] measured muscle glycogen concentrations in arm and leg biopsy samples, while using changes in body mass, body water derived from a tritium dilution, and muscle mass derived from potassium measurements to estimate a glycogen to water ratio ranging from 1:3 to 1:5. Caveats noted by the authors included the uncertainties of the measurements and the inability to measure the site of the water storage [38]. An updated version of this study, using bio-electric impedance (BIS) to measure body water and MRS to measure muscle glycogen, calculated an increase in intra-cellular water that aligned with a 1:4 ratio [39]. Despite modern techniques, issues related to the precision of measurement and the nature of the increase in body water remain. Furthermore, these studies have involved conditions in which fluid availability was optimised while muscle glycogen stores were manipulated.

Various scenarios can occur in which tissue water changes independently of changes in glycogen stores. Indeed, ultrasound technology was proposed as a technique to monitor tissue hydration in athletes [40], particularly as a marker of dehydration in athletes in weight-making sports [41]. Early understandings of muscle glycogen synthesis theorised that the associated water storage might play a regulatory role in this process. However, a study of post-exercise muscle restoration over a 15 h period found that cyclists who were dehydrated by ~5% BM or 8% body water had similar glycogen synthesis, but lower muscle water content than the trial in which they were euhydrated during recovery [42]. Meanwhile, Fernandez-Elias et al. investigated changes in the glycogen and water content of muscle samples collected over 4 h of recovery from strenuous exercise, reporting a ratio of 1:3 when the subjects were dehydrated (replacing only 400 mL fluid) and 1:17 when a volume equal to the total fluid deficit (~3170 mL) was consumed [43]. It was noted that these calculations included all water in the muscle rather than that bound to the glycogen.

Other muscle solutes, including elements that can be acutely changed, contribute to its osmotic environment. It is well documented that rapid creatine supplementation protocols are associated with an increase (~1 kg) in body mass that is largely attributed to a gain in body water [44,45,46]. Results from the larger study from which the Bone MuscleSound^®^ data were collected included a 6% increase in muscle creatine concentrations and a 22% increase in muscle glycogen when their respective loading protocols were undertaken according to best practice principles [29]. The corresponding changes in total body water and intracellular water, measured via BIS, were 1.3% and 1.4% (creatine loaded), and 2.3% and 2.2% (glycogen loaded), respectively [29]. It is possible, therefore, that changes in muscle creatine, and its associated effect on muscle water, contributed to failure of the MuscleSound^®^ to accurately track the changes in muscle glycogen stores. Indeed, we showed that these changes in muscle water, creatine, and glycogen confounded the measurement of body composition via dual X-ray absorptiometry in this study, due to a violation of the assumptions of normal relationships between these body characteristics [29].

In summary, the presence of a consistent relationship between muscle glycogen and water is not supported due to plentiful evidence that many factors, which occur frequently within sport, can independently manipulate either or both features. Theoretically, even if the MuscleSound^®^ technique was successfully calibrated to measure muscle glycogen against a specific glycogen:water content in specific conditions, it will be invalid under conditions in which this specific ratio is not present. Although further studies that accommodate these different conditions may help to enhance the algorithms linking ultrasound images to a glycogen measurement, the large number of potential scenarios that require investigation is likely to make this process difficult to achieve and incorporate into calculations. In the absence of such information, it is difficult to confidently identify scenarios in which the assumptions underlying the current MuscleSound^®^ technique might be valid. Although such conditions were not explicitly explored or identified in published literature on the MuscleSound^®^ technique, the position stand on the company website identifies conditions under which its use is optimal and sub-optimal (Table 2). Such conditions appear to overlap and to cover some, but not all, of the scenarios previously identified in which glycogen to water ratios might be altered.

### 6.2. Specific Criticism of Studies That Fail to Support the Validity of MuscleSound^®^

Data sets in which a MuscleSound^®^ assessment of muscle glycogen content failed to track the measurements achieved by chemical analysis of biopsy-derived muscle were criticised on the basis that variables that interfered with the water balance of the muscles were introduced. Concerns were raised regarding the study of the rugby league match, noting that the study methodology described data collection as “occurring within 40 min of the finish of the game”. It was asserted that such a period could have allowed the presence of artefacts, such as the effects of muscle microtrauma from the game activities, post-exercise glycogen synthesis from lactate, and a lack of control of fluid intake during the recovery period [12]. Support for these statements was provided from studies which observed fluid shifts when >3 L of fluid was consumed over 4 h of recovery [43], or low rates of glycogen synthesis (1–2 mmol/kg ww/h) in recovery from high-intensity exercise in the absence of carbohydrate intake [47]. However, it was also noted that the recently published study of glycogen use in a soccer match failed to describe the post-exercise assessment, other than that it was “immediately” after the game. No information was provided about hydration status prior to the match nor fluid intake during the match in this study, although other investigations of elite soccer players have noted that individual players may commence a match in various states of fluid balance, including significant dehydration, and incur variable rates of sweat loss during a match [48,49]. Therefore, it is curious to propose differences in tolerances to such potentially confounding factors between essentially similar studies. Although the presence of some confounding factors was acknowledged in both studies, it was noted that if the MuscleSound^®^ technique was to be promoted for use in real-life sport, it needs to be sufficiently robust to tolerate the practical conditions of use (a likely short interval between the cessation of exercise and access to each athlete to undertake assessments).

The cycling protocols involved in the Bone study (presented here) and the investigations by Routledge et al. [9] adhered to the optimal scenarios for use of MuscleSound^®^ assessments and included control around fluid intake and status. We identified that creatine supplementation may cause a change in glycogen stores and muscle water content; this formed the basis for our interest in undertaking the study of MuscleSound^®^ under such conditions. However, this technique failed to detect a difference in the glycogen assessments between the creatine and placebo groups for any treatment, and failed to detect differences between the baseline and depleted treatments for the total group of participants before the creatine supplementation commenced. Therefore, it does not appear to provide a sole or major artefact explaining the failure of the MuscleSound^®^ technique to assess muscle glycogen content in our study.

## 7. Validity of the MuscleSound^®^ Technique: Location of the Muscle Site

### 7.1. General Principles

The two original validation studies of the MuscleSound^®^ technique [4,5] used protocols that allowed the biopsy to be taken from the identical site on which the image was captured. Meanwhile, as previously identified (Table 1), the outlying studies ([9] and the Bone study presented here) took care to standardise the sites from which both ultrasound images and biopsy samples were collected, but used different sites from the same muscle between and within treatments to accommodate best practice associated with the collection of sequential muscle biopsies. A key premise of the MuscleSound^®^ protocol, at least in research scenarios, was that the location of the image for sequential assessments or comparison with biopsy assessments must be identical. However, much of the extended commentary about the protocol [10], and the specific criticisms of studies which found it did not provide a valid assessment of muscle glycogen [12], misunderstood or misrepresented the larger literature on the assessment of muscle glycogen. Specifically, comments about the variability of glycogen within muscle [10,12] demonstrated a failure to understand the capability of various assessments techniques.

This review has identified that all studies of muscle glycogen in humans utilised indirect and sampling techniques. The basis of such sampling, which occurred in both of the original MuscleSound^®^ validation studies, is that a small piece of muscle collected in a biopsy needle or captured in an ultrasound image includes sufficient muscle fibres to represent the aggregated features of individual fibres. Indeed, both the MuscleSound^®^ score and the chemical analysis of a biopsy sample represent the characteristics of “mixed muscle”. Enhanced techniques of analysing biopsy samples include histochemical staining techniques to identify differences in the storage and utilisation of glycogen between different types of muscle fibres [50], and, more recently, electron microscopy of single fibres identified different sub-cellular locations of glycogen particles [21,22,51,52]. Such techniques have helped to understand exercise metabolism and mechanisms of fatigue during exercise. However, ultrasound techniques, just like chemical assessments of homogenates or mixed muscle samples, cannot achieve such a granular assessment. Rather, they provide an overarching, yet still valuable, perspective of muscle fuel stores. In the case of muscle biopsies, there is a specific reason to require and validate the use of different sites for sequential biopsy samples; subsequent biopsies need to be taken from muscle that has not had its glycogen storage capacity impaired by trauma from the first biopsy [19]. However, it was shown that differences in the glycogen content of mixed muscle samples, representing the average of a large number of individual muscle fibres from a number of individual sites across a muscle, are minor [23]. We acknowledge that the collection of biopsy samples from adjacent, but non-identical sites, or sites from contra-lateral limbs in scenarios involving symmetrical exercise protocols, may contribute to the technical error of measurement involved with chemical determination of muscle glycogen stores. Nevertheless, it is the basis of a robust literature involving many hundreds of studies, which have determined resting muscle glycogen concentrations in different populations [36], glycogen utilisation during exercise [1,53], and glycogen synthesis in response to diet [19,54,55].

Although the size of a biopsy sample can be measured (typically, 20–200 mg), the size and location of the actual site captured in the ultrasound image is uncertain. Since the analysis of the scan is undertaken via proprietary cloud-based software, the precise size and location of the sample and site, and its ability to represent the total muscle, ultimately lies with the company software, rather than the scan technician. Nevertheless, even if there are concerns about the validity of MuscleSound^®^ glycogen assessments, there is some evidence of its reliability in estimating glycogen content across a muscle site. Indeed, the first study of the technique noted a significant correlation between the MuscleSound^®^ glycogen content and its changes due to an exercise bout between two *separate* muscles. Here the correlation between glycogen stores of the rectus femoris and vastus lateralis were *r* = 0.93 (*p* < 0.0001), *r* = 0.91 (*p* < 0.0001), and *r* = 0.76 (*p* < 0.0001) for the pre-exercise, post-exercise, and exercise change scores, respectively [4]. Furthermore, in the Bone study reported in this review, we found a significant correlation between MuscleSound^®^ scores at a single site and a shifting site within the same muscle, across a range of treatments (Figure 3). Therefore, in theory and in practice, there is evidence that in the absence of muscle damage, changes in glycogen in response to diet and exercise are similarly expressed across the gross aspect of a muscle.

### 7.2. Specific Criticism of Studies That Fail to Support the MuscleSound^®^ Technique

The major rebuttal of data sets that have found that the MuscleSound^®^ technique was unable to provide a valid measurement of muscle glycogen [10,12] is that “since glycogen is stored in different pools within a same muscle and therefore not uniformly stored, technically it is not possible to correlate the glycogen content from a very small portion of a muscle (1–2 cm^2^) with the glycogen content of an entire muscle” [10]. We have identified that this criticism of mixed muscle samples was confused with findings of sub-pools of glycogen within a single muscle fibre or between fibre types, and does not provide a legitimate understanding of broader muscle glycogen assessment. It is not necessary to make further comment on this issue. Nevertheless, if differences in the glycogen content of different sites within the same muscle do exist, this might provide an explanation for the lack of correlation between the biopsy site and ultrasound site in the dissenting studies discussed within this review. However, it fails to explain the failure of the MuscleSound^®^ protocol to detect changes in its glycogen assessment metrics when across ultrasound scans taken at the same site on subsequent occasions. That such differences were both logical, based on knowledge of supervised diet and exercise treatments, and easily detected from chemical analysis of biopsy samples creates legitimate concern about the validity of the MuscleSound^®^ technique.

## 8. Conclusions

We acknowledge the exciting potential and value of having a relatively inexpensive, portable, and non-invasive method to measure muscle glycogen in sports nutrition research and practice. Furthermore, we note that ultrasound techniques may provide new roles in sports nutrition, such as in the assessment of body composition. However, careful analysis of the literature, including previously unpublished results of our own study, fails to provide clear support for the use of an ultrasound technique (MuscleSound^®^) to measure muscle glycogen content or its changes due to supervised exercise and dietary treatments. This may be both a problem of the underlying principles of the technique, as well as the failure of currently available algorithms to cover a larger range of changes in muscle glycogen, or other manipulations of muscle solutes and water, than are often seen in sports nutrition practice. We acknowledge that two validation studies have reported the apparent success of this technique in assessing changes in muscle glycogen in similar scenarios of diet and exercise. Notwithstanding these data, the validity of the use of this technique to assess muscle glycogen, especially in field uses where conditions and treatments may be less controlled then that achieved in research situations, must be considered equivocal. Further independent studies are warranted and should include a variety of scenarios in which muscle glycogen is manipulated across the range of concentrations commonly seen in athletes, with or without changes in muscle water and solute content. Interrogation of laboratory and real-world scenarios should also be included to investigate the tolerance of this method to differences in the logistics and rigour of data capture.

## Figures and Tables

**Table 1:** Studies involving ultrasound (MuscleSound^®^) measurements of muscle glycogen, including comparison to biopsy-derived chemical assessments of glycogen.

	Hill & San-Millan 2014	Nieman et al., 2015	Routledge et al., 2019a	Routledge et al., 2019b	San-Millan et al., 2020	Bone et al., 2020
Study population	22 M cyclists (competitive: professional and amateur: category 1–4)	20 M cyclists (regular competitors in road and TT: VO_2_max: 47.9 ± 7.8 mL/kg/min)	14 M rugby league players (professional)	16 M recreationally active VO2max 49.9 ± 7.5 mL/kg/min	9 M soccer players: (professional: U.S. major soccer league)	12 M cyclists/triathletes (well-trained: VO2max 64.5 ± 7.6 mL/kg/min)
Scenarios of glycogen measurements	Pre-exercise: CHO loaded Post exercise depletion (endurance cycling)	Pre-exercise: normalised glycogen? Post exercise depletion (endurance cycling)	Pre-exercise: normalised glycogen? Post-exercise depletion (field: team sport)	Pre-exercise: normalised glycogen Post-exercise: substantial depletion CHO loaded	Pre-exercise: normalised glycogen? Post exercise depletion (field: team sport)	Pre-exercise: normalised CHO Pre-exercise: maximally CHO loaded Pre-exercise: normalised CHO + creatine loaded Pre-exercise: CHO loaded and creatine loaded Post-exercise: substantial deletion
Dietary protocols (CHO intake)	Glycogen preparation: “optimized” via 3 days @ 8 g/kg Pre-exercise meal: NA During exercise: NA (self-managed with instructions)	Glycogen preparation: NA Pre-exercise meal: NA During exercise: water only	Glycogen preparation: NA Pre-exercise: NA During exercise: water only	Supervised exercise depletion followed by either 36 h of low CHO (2 g/kg) or high CHO (8 g/kg)	Glycogen preparation: “24 h team nutrition protocols”; pre-exercise meal: “team nutrition”; during exercise: 40 g at warm up and 65 g at half time	Glycogen preparation: Normalised: 48 h @ 6 g/kg CHO loaded: 48 h @ 12 g/kg Pre-exercise meal: 2 g/kg During exercise: 60 g/h Depletion: 18 h @ 1 g/kg
Exercise protocol	90 min cycling on lode ergometer at “moderate-high intensity eliciting CHO oxidation rates of 2–3 g/min.”	75 km (~168 min) TT on own bike mounted on ergometer	80 min rugby league match	Glycogen depletion cycling protocol: 90%/50% PPO and 80%/60% PPO until exhaustion (* low CHO trial: extra 45 min at 60% PPO).	90 min soccer match.	120 km TT (alternating 1 and 4 km sprints every 10 km) + TTE on cycle at 8% gradient and 88% VO_2_max.
Timing of glycogen measurements	Baseline: immediately before exercise Post-exercise: NA	Baseline: NA Post-exercise: within 20–30 min	Baseline: 60 min pre-match. Post-exercise: within 40 min.	Post-exercise: NA Depleted: 36 h after exercise + low CHO CHO loaded: 36 h after exercise + high CHO	Baseline: 10 min before warm-up. Post-exercise: within 5–10 min	Pre-exercise: 2 h prior to exercise (e.g., before pre-exercise meal) Depleted ~18 h post exercise + low CHO
Muscle assessed	Rectus Femoris (U and Bx) U on Vastus lateralis: (data not provided)	Vastus lateralis Rectus Femoris	Vastus lateralis	Vastus lateralis	Rectus femoris (U only)	Vastus lateralis
Muscle state	Contracted	Not advised	Relaxed	Relaxed	NA	Relaxed
Muscle biopsy location	Baseline Bx on right leg. Post-exercise Bx on left leg. Mid-point between ASIS to superior patellar. Same location as ultra-sound	Baseline and post-exercise Bx on same leg 2 cm apart Same location as ultrasound	Baseline and post-exercise Bx on same leg 2 cm apart	Bx for low and high CHO dietary conditions on same leg 1–2 cm apart	Nil	Bx on same leg. Mid-point between ASIS and anterior superior aspect of patella. Four sites 2 cm apart (see Figure 1)
Ultrasound location	Same leg as Bx. Baseline U on right leg. Post-exercise U on left leg	Same leg as Bx	Same leg as Bx. 50% of length and width of VL determined by U	Same leg and same site as Bx. 50% of length and width of VL determined by U	NA—same leg used for pre- and post-U scans?	U on contralateral leg (1) at corresponding location to muscle biopsy (at each of 4 sites—Figure 1)
Ultrasound Scan protocol	NA	Mean of 3 scans	NA	NA	Mean of 2 scans	Mean of 5 scans
Glycogen data *	Bx: Glycogen (mmol/kg dw) reduced from 416 ± 146 to 267 ± 98 by exercise (*p* < 0.001). U: MuscleSound^®^ score (a.u.) reduced from 59.8 ± 15.9 to 39.8 ± 13.9 post-exercise (*p* < 0.0001)	Bx: Glycogen (mmol/kg dw) showed mean change of 306 ± 99 * due to exercise (~407 to 101) (*p* < 0.001). U: baseline and post-exercise glycogen score data not provided	Bx: Glycogen (mmol/kg dw) reduced from 443 ± 65 to 271 ± 94 (*p* < 0.0001) by exercise. U: no change in MuscleSound^®^ score from baseline (47 ± 6 a.u.) to post-exercise (49 ± 8 a.u.; *p* = 0.4)	Bx: Glycogen (mmol/kg dw) with high dietary CHO: 531 ± 129 vs. low CHO dietary intake: 252 ± 64 (*p* < 0.001). U: MuscleSound^®^ score (a.u.) with high dietary CHO: 56 ± 7 vs. low dietary CHO intake: 54 ± 6 (*p* = 0.3)	Bx: No biopsy conducted. U: MuscleSound^®^ score (“points”) decreased from 80 ± 8.6 to 63.9 ± 10.2. (*p* = 0.005)	Bx: Glycogen (mmol/kg dw) reduced from 639 ± 115 to 276 ± 115 with depletion and increased with CHO loading to: 730 ± 98 (*p* < 0.05) U: MuscleSound^®^ score (a.u.): 55 ± 10 (baseline); 52 ± 13 (depletion) and 56 ± 8 (CHO loaded), NS. U: EFL (points): 79 ± 18 (baseline); 70 ± 22 (depletion) and 90 ± 14 points (CHO loading), NS.

M, male; a.u., arbitrary units; Bx, biopsy; U, Ultrasound scan; ASIS, anterior superior iliac spine; @, at; CHO, carbohydrates; VO_2_max, maximal oxygen capacity; NA, not available; PPO, peak power output; NS, not significant; and EFL, estimated fuel level; * All biopsy-derived glycogen values presented as mmol/kg dry weight (dw), with conversion from mmol/L wet weight (ww) involving multiplication by 4.28 [34].

**Table 2:** Scenarios of use of MuscleSound^®^ measurement of muscle glycogen *.

Optimal Scenarios	Sub-Optimal Scenarios
Pre and immediately post-exercise Several hours after the end of moderate to high intensity/long duration exercise (such as cycling that does not involve extensive eccentric contractions) One to two days or more after high intensity/long duration sports such as soccer, football, rugby, and basketball One to two days before a competition	Within several hours of the end of moderate to high intensity/moderate duration steady state exercise The day after high intensity/long duration competition in sports such as soccer, football, rugby, and basketball

* Information taken from MuscleSound^®^ position stand on Science and Application [13].

## Data Availability

The full data set reported in this study will be made available upon request to the corresponding author.
